# HiCommunication as a novel speech and communication treatment for Parkinson's disease: A feasibility study

**DOI:** 10.1002/brb3.2150

**Published:** 2021-05-04

**Authors:** Ellika Schalling, Helena Winkler, Erika Franzén

**Affiliations:** ^1^ Division of Speech and Language Pathology Department of Clinical Science Intervention and Technology Karolinska Institutet Stockholm Sweden; ^2^ Medical Unit Speech and Language Pathology Karolinska University Hospital Stockholm Sweden; ^3^ Division of Physiotherapy Department of Neurobiology, Care Sciences and Society Karolinska Institutet Stockholm Sweden; ^4^ Medical Unit Occupational Therapy and Physical Therapy Karolinska University Hospital Stockholm Sweden

**Keywords:** communication, feasibility, intervention, Parkinson's disease, speech

## Abstract

**Introduction:**

Speech and communication problems are common in Parkinson's disease (PD) and can result in social withdrawal and reduced quality of life. Intervention may improve symptoms but transfer and maintenance remain challenging for many. Access to treatment may also be limited. Group intervention incorporating principles for experience‐dependent plasticity may address these challenges. The aim of this study was to develop and study feasibility aspects of a new intervention program for group training of speech and communication in people with PD.

**Materials & Methods:**

Development and content of the program called HiCommunication is described. Core target areas are voice, articulation, word‐finding and memory. Five participants with mild‐moderate PD completed this feasibility trial. Attendance rate and possible adverse events as well as the participants' experiences were documented. A speech recording and dysarthria testing were completed to study feasibility of the assessment procedure and evaluate possible changes in voice sound level and intelligibility.

**Results:**

Attendance rate was 89%. No adverse events occurred. Participants reported a positive experience and limited fatigue. Assessment was completed in approximately 30 min and was well tolerated. Four of five participants had an increased voice sound level during text‐reading postintervention and mean intelligibility improved.

**Conclusions:**

Results indicate that HiCommunication is feasible for people with mild‐moderate PD. The program was appreciated and well tolerated. Positive outcomes regarding voice sound level and intelligibility were observed; however, the number of participants was very limited. The results motivate that effects of HiCommunication are further studied in a randomized controlled trial, which is ongoing.

## Graphical abstract

The aim of this study was to develop and study feasibility aspects of a new intervention program for group training of speech and communication in people with Parkinson's disease called HiCommunication. Results indicate that HiCommunication is feasible for people with mild‐moderate PD, well‐tolerated and positive outcomes following intervention were shown.

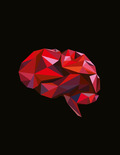

## 1 INTRODUCTION

Parkinson's disease (PD) is one of the most common neurodegenerative progressive diseases. Prevalence in individuals world‐wide rises with age, from 40/100,000 individuals in ages 40–49 years to as high as 1,903/100,000 in ages 80 and older (Pringsheim et al., 2014). Gross motor symptoms, caused by underlying dopaminergic deficiency include bradykinesia, tremor, rigidity and postural instability (Armstrong & Okun, 2020). In addition, many people with PD (PwPD) suffer from nonmotor symptoms such as cognitive impairment, psychiatric symptoms and sleep‐disorders to name some of the more common problems (Zis et al., 2015). Impaired executive functions such as difficulties with attention and dual‐task performance, as well as memory changes are examples of common cognitive deficits in PwPD (Dirnberger & Jahanshahi, 2013).

Speech and voice function are affected in up to 90% of PwPD (Hartelius & Svensson, 1994; Logemann et al., 1978). Symptoms primarily include changes in the areas of voice, articulation and prosody and are summarized under the term hypokinetic dysarthria. Common characteristics are reduced loudness, hoarse and/or breathy voice quality, monotone pitch, imprecise articulation and increased or variable speech rate (Darley et al., 1969). In addition, central sensory processing abnormalities as well as impaired internal cueing result in reduced awareness of the sound level of the own voice and difficulties with self‐regulating adequate loudness (Clark et al., 2014; Ho et al., 2000; Kwan & Whitehill, 2011). Possibly attentional deficits also play a role in difficulties with maintaining adequate loudness in conversation (Liu et al., 2019). Speech symptoms often present quite early in the disease process, contrary to findings in older studies (Rusz et al., 2011, 2013; Rusz, Hlavnička, et al., 2016. Progression of speech symptoms commonly leads to reduced communicative participation with negative effects on quality of life (Miller et al., 2006, 2008; Schalling et al., 2017).

Pharmacological intervention generally has a positive effect on motor symptoms, at least in the earlier phases of the disease development, whereas treatment response for axial symptoms such as speech impairment is more variable (Rusz, Tykalová, et al., 2016; Pinto et al., 2004). Deep‐Brain Stimulation (DBS) sometimes has limited effects regarding speech symptoms and can even result in deterioration of speech function (Aldridge et al., 2016). The variable and sometimes negative response to pharmacological and surgical intervention motivates the need for other approached to alleviate speech symptoms and counteract possible subsequent negative consequences for communication.

Behavioral treatments for speech symptoms in PD have traditionally focused on one, or a combination of different aspects of speech production via exercises targeting respiration, phonation, articulation or prosody. During the last two decades, the Lee Silverman Voice Treatment (LSVT‐LOUD^®^) has become a widely used and studied intervention program. LSVT‐LOUD^®^ primarily focuses on increased vocal loudness by targeting the respiratory‐laryngeal subsystem of speech production. Secondary gains such as improved articulatory precision contributing to increased intelligibility have also been shown as a result of increased respiratory and phonatory effort (Ramig et al., 2004; Dromey et al., 1995; Sapir et al., 2007). The intervention also addresses the need for recalibration of effort to accomplish adequate loudness levels. LSVT‐LOUD^®^ is an intensive program of 16 sessions over 4 weeks including daily home exercises (Ramig et al., 2007). Several randomized controlled trials (RCT) have demonstrated significant improvement of voice sound level, in one study up to 24‐month postintervention (Ramig et al., 1995, 2001). In a more recent and larger RCT including 84 participants positive changes of voice sound level and on a modified Communication Effectiveness Index (CETI‐M) for the participants in the active treatment group were demonstrated in comparison to control treatment or no treatment at one‐ and 7‐month postintervention (Ramig et al., 2018). A significant improvement of intelligibility was also shown in the group of participants who received LSVT‐LOUD^®^, whereas there was no statistically significant change for participants who received articulatory training and a decrease for the group that got no training (Levy et al., 2020). Although it can be concluded that LSVT is a treatment with documented positive effects for PwPD, it is an intervention demanding resources both in terms of staff availability and patient stamina, and it may not be available for all suitable candidates. Alternative modes of delivering LSVT‐LOUD^®^ have been developed to address problems with accessibility (Spielman et al., 2007; Halpern et al., 2012). The intensity may, however, still be a limiting factor for people with many motor and nonmotor symptoms, severe fatigue or difficulties with transportation to a clinician far away four times/week. Limited resources, factors related to organization of the health care system and lack of LSVT‐certified clinicians may also be issues limiting accessibility in some parts of the world.

Effect of treatment is usually evaluated by analyses of speech recordings. It has, however, been shown that data from studio recordings may not be representative of voice use in everyday life where other factors such as stress, cognitive loading and fatigue may divert attention from maintaining a good voice technique acquired in treatment (Gustafsson et al., 2019a). Clinical experience also supports the impression that the major challenge for PwPD with speech symptoms who undergo speech‐language pathology intervention is to transfer skills acquired in treatment to situations in everyday life. Other aspects that have come forward in interviews with PwPD about their experiences of intervention is a desire to incorporate more focus on interpersonal communication and interaction with others in the treatment of communication difficulties (Yorkston et al., 2017).

Challenges related to transfer of treatment effects to communicative situations outside the clinic, reduced access to individual treatment and limited ability to participate in intervention four times/week in the clinic have motivated development of a new program for group treatment to improve speech and communication in PwPD. Based on principles of importance for experience‐dependent neural plasticity believed to promote relearning of motor behavior such as intensity, repetition, specificity and saliency (Kleim & Jones, 2008), we acknowledged the need for a group‐based, speech and communication intervention to improve voice intensity and articulatory precision used in communicative situations outside the clinical setting.

The aim was therefore to develop a group treatment, HiCommunication, to address speech and communication difficulties for PwPD, incorporating principles for experience‐dependent plasticity. The intent was also to take cognitive challenges facing PwPD as well as their desire to participate in intervention with stronger focus on psychosocial interaction into account. In this present study development of the program was described and feasibility aspects related to process such as recruitment, attendance, data collections tools and acceptability as well as scientific aspects such as treatment safety and indications of effect were studied.

## 2 MATERIALS AND METHODS

### 2.1 Design

In this development and feasibility study, a new exercise regime for speech and communication in PwPD is described and tested in a small pilot format to assess aspects of feasibility before evaluating the effects of the intervention in a large randomized controlled trial (ClincalTrials.gov: NCT03213873).

### 2.2 Development of the HiCommunication program

The new group treatment program called HiCommunication was developed in collaboration with a group of Speech‐Language Pathologists (SLPs) with long experience of treatment of speech and communication in PD. The practicing clinicians were invited to give feedback on content and format of the intervention during development of the program. The SLP who delivered the intervention in this pilot feasibility study was especially active and also contributed to completing the bank of exercises and the book for home‐training used in HiCommunication. In addition, a focus group interview with PwPD was conducted in the early phase of development of the program. The participants shared their experiences from previous speech‐language pathology intervention, their priorities and what they had appreciated in previous experiences of intervention. Another source of inspiration was a group‐training program for balance and gait for PwPD developed by physiotherapists (Conradsson et al., 2014, 2015). This physiotherapy program was based on principles considered to promote neuroplasticity and had a progressive structure with four core areas related to balance in focus (Conradsson et al., 2012). HiCommunication was also inspired by concepts based on principles of motor learning in that the training is relatively intensive with multiple repetitions of target behaviors, specific key aspects of speech production are practiced and exercises are performed in a communicative context with materials of interest to participants to increase saliency. The core areas targeted in HiCommunication are voice intensity and articulatory precision, the objective being to obtain loud and clear speech (Kearney et al., 2017; Tjaden et al., 2013, 2014). Word‐finding and memory are two other core areas, however, mainly incorporated into the program to progressively increase cognitive loading during speech exercises (Cholerton et al., 2014). Incorporating gradually more complex content while practicing newly acquired speech technique is included in the training to resemble the challenge PwPD face in communicative interaction where focus must be on both content and manner of speaking to successfully overcome speech constraints. The idea is that repeated exercises using this “dual‐task” format during controlled training sessions with gradually fading support will enhance transfer of improved speech technique to situations outside the clinical context. See Table 1 for a summary of core areas, objectives and exercises in the HiCommunication program.

**TABLE 1:** Core areas of intervention to meet constraints of speech and communication in Parkinson's disease (PD) and exercises to meet objectives of the intervention

Core area	Constraints in PD	Objective	Exercises designed to reach objective
Voice intensity	Reduced voice intensity Reduced awareness of soft voice Difficulties self‐regulating voice intensity	Maintain adequate voice sound level in communicative situations	Use loud voice with good voice quality in exercises with increasing complexity and with increased cognitive loading (see below)
Articulatory precision	Imprecise articulation	Maintain clear speech in communicative situations	Use good articulatory precision in speech exercises with increasing complexity and with increased cognitive loading (see below)
Word‐finding	Impaired word‐finding Reduced verbal fluency	Improved verbal fluency in conversation	Naming and association tasks (e.g., to find synonyms, antonyms, define homonyms, associate noun to verb) Verbal fluency tasks, (e.g., to produce words in a semantic category)
Memory	Impaired memory function	Improved memory function in communicative situations	Memory games (e.g., recall wordlists of increasing length, memorize collection of objects, reiterate stories)

HiCommunication is a 10‐week program with a total of 30 hr of training. Each participant completes two 1 hr‐long sessions in a group of 5–7 PwPD led by an SLP, and one session of self‐practice at home supported by a training diary. The decision to include 1 weekly home‐session was based on the clinical experience that many PwPD find it too challenging to travel to the clinic three times/week, and as a compromise in order to limit healthcare resources. A group size of 5–7 individuals is considered optimal in that it gives opportunities to practice in pairs or small groups of three participants and thus providing mass‐practice also in a group format, but it is still a small enough group that one clinician can lead the session, adapt exercises and give individual feedback to the participants. SLPs delivering the HiCommunication intervention should have knowledge about PD and its limitations on voice and communication. They also need specific training to learn the format and content of the program before initiating the training.

### 2.3 Program progression

Level of challenge and complexity of the training progresses steadily over the 10 weeks. Better speech technique is initially established in simple exercises and the improved speech technique is then applied in communicative activities with other participants in the group. Cognitive loading is gradually increased through the program by adding more complexity to tasks and the communicative situations while the aim is to maintain the improved speech technique. The theoretical reasoning is to improve dual‐task capacity, to enhance use of improved speech technique while cognitive resources are also needed to process content of the tasks or interaction with the communication partner. The progression of training is achieved by organizing the program into three exercise blocks, see Table 2. During block A focus is on simple exercises to master use of increased voice intensity and improved articulatory precision. The concepts loud and clear (relating to voice intensity and articulatory precision) are introduced and practiced in simple phonatory exercises and in words and short phrases. Loud and clear are first targeted separately and then in combination. During block B cognitive loading is increased by starting to use loud and clear speech in more complex speech tasks and in interaction with other group participants. During this phase word‐finding and memory tasks are also introduced and participants are prompted to maintain loud and clear speech during all the exercises. During block C the cognitive loading is further raised by demanding more interaction between participants and by adding distractors such as background noise during exercises or by completing exercises in other environments such as the hospital hallway or cafeteria.

**TABLE 2:** Description of the intervention (HiCommunication)

Program format	30 session over 10 weeks 2 sessions/week in group with SLP 1 home‐training session with training diary
Personnel	One speech and language pathologist (SLP)
Setting	University hospital Room for group treatment in SLP clinic
Core areas	Voice intensity Articulatory precision Word finding Memory
Block A, weeks 1–2	Improving speech technique The concepts “loud” (increased voice intensity) and clear (speech with increased articulatory precision) are introduced and practiced Exercises include breathing, sustained loud phonation, articulation of words and phrases with increased voice intensity and articulatory precision while maintaining good voice quality
Block B, weeks 3–6	Continued practice of loud and clear speech in exercises with increased complexity including short dialogues or narratives. Increased cognitive loading in exercises is introduced by adding word‐finding tasks, memory games and association tasks
Block C, weeks 7–10	Continued practice of loud and clear speech with increased cognitive loading. Speech tasks with demanding more interaction between participants, and more complex exercises are introduced while maintaining loud, clear speech. Increased demands also by adding background noise or moving to settings outside the treatment room
Home exercise program, performed once a week with training diary	Relaxation and breathing exercises Voice and speech exercises Word and memory exercises

### 2.4 Participant recruitment

Participants were recruited via advertisement, via the Swedish PD Association and via clinical referrals from the speech‐language pathology clinic at the university hospital.

Inclusion criteria were PD diagnosed by neurologist, Hoehn and Yahr (1967) stage 2–3, age ≥60 years and a score of ≥ 21 on Montreal Cognitive Assessment (MoCA) (Nasreddine et al., 2005). Exclusion criteria were other neurologic or psychiatric diseases affecting speech and communication and participation in speech‐language pathology intervention in the last 6 months. Additional exclusion criteria were magnetic resonance imaging (MRI) incompatible implants or claustrophobia as the same participants also underwent structural and functional MRI as part of a related study evaluating feasibility in preparation for a larger randomized controlled trail (Johansson et al., 2020).

This feasibility study was part of a larger trial approved by the Regional Ethical Review Board in Stockholm 2016/1264‐31/4, 2017/1258‐32 and 2017/2445‐32. The participants received written and oral information about the study and all its assessment and provide written informed consent before the start of the assessments.

Candidates were screened for eligibility via an initial telephone interview and then assessed for inclusion. During the first treatment session, the SLP perceptually assessed the participants' quality of voice. If there was any suspicion of laryngeal dysfunction of other etiology than PD, the participant was examined by a phoniatrician before the next session to exclude the risk of causing damage by voice exercises in case of an underlying undiagnosed organic voice disorder.

### 2.5 Assessments

Assessment was performed in the on stage of medication, within 3 weeks pre and postintervention. Testing included a speech recording according to a standardized protocol including maximum sustained phonation, syllable repetition, reading of sentences and a paragraph and monologue. Speech recordings were completed in a sound‐proof recording studio with a Sony Digital Audio Tape Deck DTC‐ZE700. The participants wore a microphone (Sennheiser HSP 4 with a MZA 900 P phantom power adapter), mounted on a headset to guarantee a constant mouth‐to‐microphone distance during recordings.

Dysarthria assessment with a standardized Swedish dysarthria test was also part of the assessment (including intelligibility testing and a self‐report questionnaire on acquired speech disorders) (Hartelius, 2015; Rusz, Tykalová, et al., 2016). Pre and postassessments were performed by an SLP who was not involved in delivering the intervention program. The external tester was also instructed to note time required for testing and any other aspects of the assessment related to feasibility. Attendance and possible adverse events were documented by the SLP leading the training. Following the intervention, the participants also filled out a questionnaire on their subjective experience of the intervention which was collected by another person than the SLP delivering treatment.

## 3 RESULTS

### 3.1 Participant characteristics

Six participants (three men and three women) were included in the pilot study. Median age was 67.5 years (range 63–70) and median disease duration was 7 years (range 3–11). Median score on MDS‐UPDRS part II was 32.5 (range 22–52) and median Levodopa Equivalent Daily Dosage (LEDD) was 765.5 (525–1171). Three participants had a Hoehn & Yahr score of 2 and three had a score of 3. Median score on MOCA was 26.5 (range 21–28).

### 3.2 Feasibility of assessment

The speech recording and dysarthria assessment was completed in approximately 30 min for all participants and no difficulties with the testing were noted. Dysarthria scores were very low or at ceiling for these participants who mainly had symptoms affecting only voice intensity and/or voice quality. One participant had some difficulties reading the text during speech recording which was resolved by providing a stimulus card with larger font size.

### 3.3 Acceptability of the program

Mean overall attendance in this pilot trial was 89.0% (*SD* 8.2). All participants reported having completed the home‐training throughout the program. One participant terminated participation after nine sessions due to medical problems unrelated to PD. No data postintervention could therefore be collected from this participant and data is therefore only reported for the five participants who completed the program. No adverse events during the training period were reported.

All five participants who completed the program reported that they would recommend this intervention to other PwPD. Three participants also agreed to a very high degree and two participants agreed to a high degree with the statement that the SLP led the group well. All five agreed to a high degree with the statement that the training had a good and useful content. There was a bit more variation in responses to the item “the training has made my voice and speech better”; two participants partly agreed, one participant agreed to a high degree and one to a very high degree (one participant did not respond to this item). The question whether other people had noticed a change in the participant's voice and speech also got more varied ratings; one participant agreed to small degree, two partly agreed, one agreed to high degree and one to a very high degree. The participants did not agree with or agreed partly with the statement that other symptoms related to PD had been affected by the intervention. Free comments in writing mentioned a sense that gait, balance and posture had improved. Three participants did not agree at all with a question about increased fatigue related to the intervention whereas two participants partly agreed with this.

### 3.4 Speech outcomes

**FIGURE 1:**
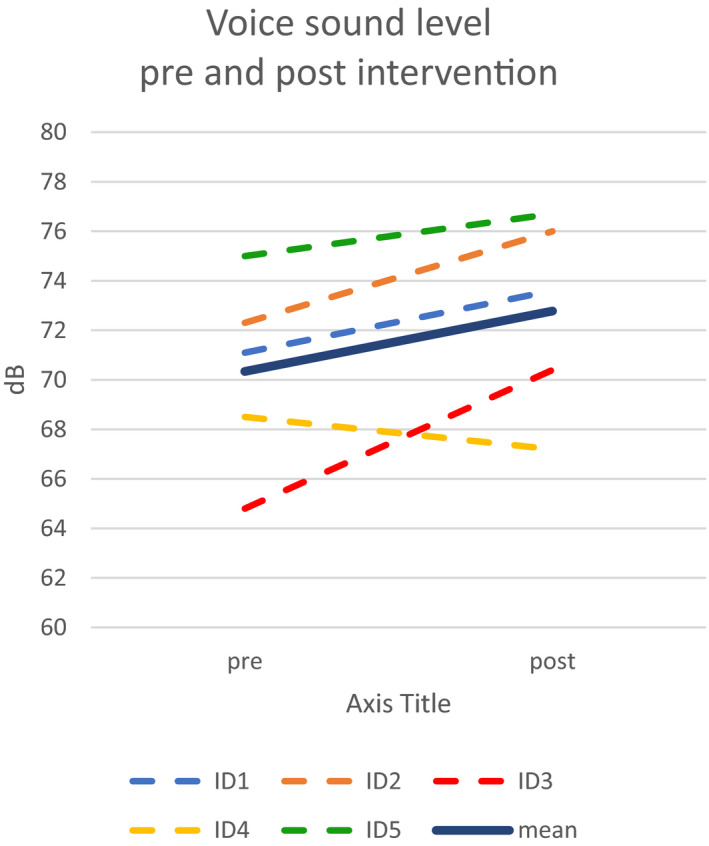
Individual mean voice sound level (dBC) during text‐reading pre and postintervention for the five participants who completed the HiCommunication program. Mean value for the five participants indicated in dark blue

Mean voice intensity increase during text reading was 2.5 dBC for the five participants who remained in the study. Changes pre and postintervention was varied and ranged between −1.3 to 5.6 dBC, see Figure 1.

**FIGURE 2:**
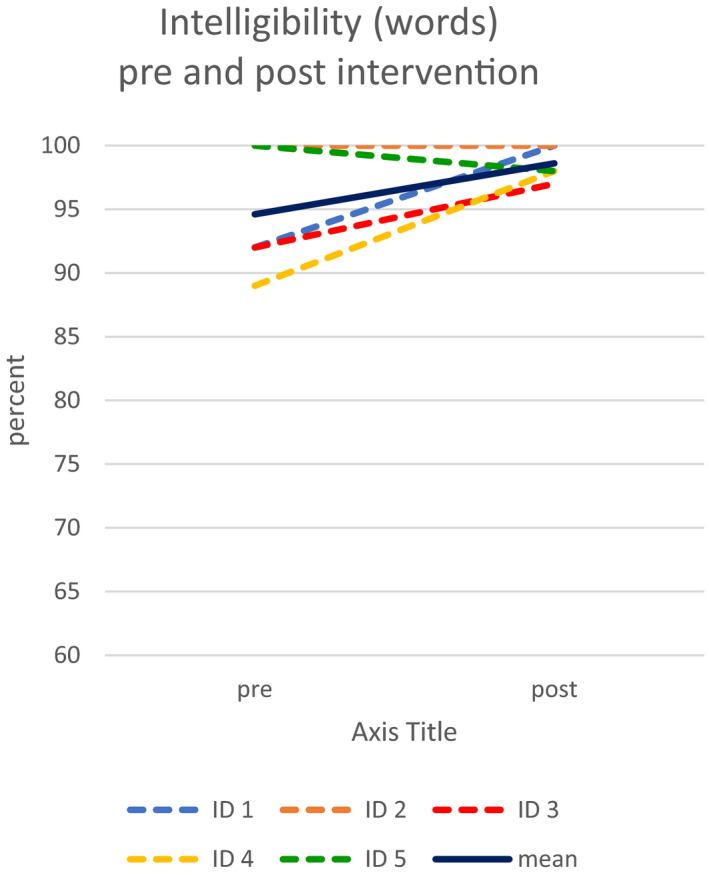
Intelligibility (words) pre and postintervention for the five participants who completed the HiCommunication program. Mean value for the five participants indicated in dark blue

Intelligibility (words) increased from an already high level of a mean of 94.6% for the five participants pre intervention to a mean of 98.6% postintervention, see Figure 2.

## 4 DISCUSSION

Results from this study indicate that the new intervention program HiCommunication for speech and communication is feasible to deliver in a university hospital setting for participants with mild‐moderate PD. The attendance rate was high, and no adverse events were reported. Unfortunately, one participant terminated participation before the intervention program was completed for medical reasons unrelated to PD. The five participants who completed the whole program stated that it was a positive experience, that they would recommend the program to others and that they perceived an improvement in speech and voice function. Some fatigue related to the training was noted by two of five participants, the other three reported no increased fatigue. An increased voice sound level was observed in four of five participants during assessment of text‐reading postintervention compared to baseline and intelligibility was also improved, although already from a high level. The assessment procedure (speech recording and dysarthria assessment) was tolerated well and the duration of approximately 30 min can be considered feasible in a clinical setting.

The small number of participants is an obvious limitation of the study and results must therefore be considered with caution. The primary aim was not to evaluate effects related to speech and communication of the program, but rather to assess feasibility in preparation for a larger RCT, ClinicalTrials.gov Identifier: NCT03213873 (Franzén et al., 2019). Results regarding both process and scientific feasibility aspects, although based on few individuals, were positive and motivated continued studies of the program in a larger trial. They high attendance supports that two visits to the clinic in combination with one self‐training session at home as well as the relatively long treatment period (10 weeks) was well tolerated. Possibly the interaction between participants in the group contributed to keep participants motivated, as was informally reported to the SLP leading the group and also included as a comment in the questionnaires following the intervention. This would be in line with positive effects of group dynamics in group training for PwPD described by (Diaféria et al., 2017). The positive effects of psychosocial interaction and doing speech exercises in communicative situations were also highlighted in an interview study with PwPD by (Yorkston et al., 2017).

The experiences of the SLP leading the group in this pilot study were also positive. She confirmed that a group size of five to six participants worked very well in that it both allowed attention to individual performance and development, while it was also possible to work in pairs or in a small group with dialogues and other exercises. In a group with too many participants, there is a risk that there is too little time for each participant to practice and get feedback but the SLP reported that there had been a good balance between an ecologically valid interaction in the group and time for the clinician to support implementation of improved voice and speech technique.

Before the pilot study, a task bank of word‐finding and memory exercises had been developed from which the SLP selected tasks for each session. A few more exercises were added to the bank based on ideas that came up during sessions, but other than that no changes of the content of to the program have been made based on experiences from the pilot study.

The SLP conducting the intervention made a perceptual analysis of the participants' quality of voice during the first session. For participant where an organic voice disorder unrelated to PD could be suspected a referral to a phoniatrician for further examination before continuing with voice exercises was made. In this feasibility trial, one participant was examined, and although no vocal fold dysfunction other than the voice problems related to PD was detected, this is a safety measure recommended also for future studies to avoid exacerbating a pre‐existing vocal fold pathology that may have been masked and undetected because of PD symptoms.

The development of the HiCommunication program was inspired by another group‐therapy intervention for PwPD aimed at improving gait and balance, called HiBalance. To allow comparison between effects of HiBalance and HiCommunication it was important to limit physical movement in the HiCommunication regime and all exercises were therefore done while seated which may also have been a limitation. Some phonatory exercises could for example have been facilitated by incorporating hand or arm gestures or other movement.

A gradual increase of cognitive loading is incorporated into the program to provide opportunities to practice maintenance of improved speech technique while also paying attention to speech content, that is, to use speech in the way speech is used in communicative situations in real life. Divided attention is known to challenge motor performance in PwPD. It has been shown that the introduction of a dual‐task exercise affects both speech volume and temporal aspects of speech production in PwPD in an experimental situation (Ho et al., 2002), a fact that is familiar to many practicing SLPs. To what degree this challenge can be alleviated by systematic training remains to be further investigated and will be addressed further in future studies of the HiCommunication program.

Changes in outcome measures relating to voice sound level (dBC) for the five participants were somewhat varied but point in a positive direction. One participant increased voice sound level with 5.6 dBC which is clearly a clinically meaningful increase, whereas one participant had a slightly lower voice sound level at assessment postintervention. The participant with the largest change was the individual with the lowest voice sound level pre intervention. The other participants had higher voice sound levels at baseline, more comparable to voice intensity in healthy speakers which may contribute to less marked changes. The increase of 5.6 dBC is comparable to mean increase of voice sound level during text‐reading from baseline to 1‐month postintervention of 6.3 dB reported in a recent RCT of Lee Silverman Voice Treatment^®^ (LSVT‐LOUD^®^) (Ramig et al., 2018). The effect on speech and communication of the intervention will be further evaluated in future studies. Another aspect that will also be addressed in future studies is to what degree positive changes for example of voice sound level were transferred to situations outside the clinical setting. This can be accomplished using an Ambulatory Phonation Monitor (APM), that is, equipment allowing long‐term registration of voice use during daily activities. It has shown that voice sound levels of PwPD and matched healthy controls from recordings in a controlled environment (such as a recording studio) are not representative of speech registered outside the clinical environment also in low background noise (Gustafsson et al., 2019a). In another recent study, comparisons of registrations between a pair of twins with similar living conditions where one twin was healthy and the other had PD and underwent LSVT‐LOUD^®^ also showed differences in voice sound level registered in daily life compared to data from registrations from a controlled registration in a studio setting (Körner Gustafsson et al., 2019b).

In conclusion, based on this first description and feasibility study, HiCommunication is a group intervention program for speech and communication for PwPD that is possible to deliver in a university hospital setting to participants with mild‐moderate PD. Future studies will continue to explore effects of the intervention.

## CONFLICT OF INTEREST

The authors declare that there is no conflict of interest.

## AUTHOR CONTRIBUTIONS

The first author ES developed the intervention described in this manuscript, in collaboration with HW and EF. The design of the study was developed by EF and ES. HW was primarily responsible for delivery of the intervention and ES was active in data collection and analyses. The manuscript was drafted by ES, but all authors read and contributed to the final version.

### PEER REVIEW

The peer review history for this article is available at https://publons.com/publon/10.1002/brb3.2150.
